# Evaluation of risk factors for perforated peptic ulcer

**DOI:** 10.1186/s12876-018-0756-4

**Published:** 2018-02-15

**Authors:** Kazuki Yamamoto, Osamu Takahashi, Hiroko Arioka, Daiki Kobayashi

**Affiliations:** 1 Division of General Internal Medicine, Department of Medicine, St. Luke’s International Hospital, Tokyo, Japan; 2 Fujita Health University, Toyoake, Japan

**Keywords:** Perforated peptic ulcer, Peptic ulcer disease, Risk factor, Case control

## Abstract

**Background:**

The aim of this study was to evaluate the prediction factors for perforated peptic ulcer (PPU).

**Methods:**

At St. Luke’s International Hospital in Tokyo, Japan, a case control study was performed between August 2004 and March 2016. All patients diagnosed with PPU were included. As control subjects, patients with age, sex and date of CT scan corresponding to those of the PPU subjects were included in the study at a proportion of 2 controls for every PPU subject. All data such as past medical histories, physical findings, and laboratory data were collected through chart reviews. Univariate analyses and multivariate analyses with logistic regression were conducted, and receiver operating characteristic curves (ROCs) were calculated to show validity. Sensitivity analyses were performed to confirm results using a stepwise method and conditional logistic regression.

**Results:**

A total of 408 patients were included in this study; 136 were a group of patients with PPU, and 272 were a control group. Univariate analysis showed statistical significance in many categories. Four different models of multivariate analyses were conducted, and significant differences were found for muscular defense and a history of peptic ulcer disease (PUD) in all models. The conditional forced-entry analysis of muscular defense showed an odds ratio (OR) of 23.8 (95% confidence interval [CI]: 5.70–100.0), and the analysis of PUD history showed an OR of 6.40 (95% CI: 1.13–36.2). The sensitivity analysis showed consistent results, with an OR of 23.8–366.2 for muscular defense and an OR of 3.67–7.81 for PUD history. The area under the curve (AUC) of all models was high enough to confirm the results. However, anticoagulants, known risk factors for PUD, did not increase the risk for PPU in our study. The conditional forced-entry analysis of anticoagulant use showed an OR of 0.85 (95% CI: 0.03–22.3).

**Conclusions:**

The evaluation of prediction factors and development of a prediction rule for PPU may help our decision making in performing a CT scan for patients with acute abdominal pain.

## Background

Although a perforated peptic ulcer (PPU) is worsened consequence of peptic ulcer disease (PUD), several clinical features and prognoses are dramatically different. One of the reasons is that symptoms of PPU vary over time and are classified into three phases. The first phase appears within 2 h of onset, and symptoms such as acute abdominal pain, tachycardia and peripheral coldness are typical of this stage. Then, a perforation releases the gastroduodenal contents into peritoneal cavity, causing chemical peritonitis. Severe pain stimulates sympathetic nerves, and tachycardia and peripheral coldness result. Within 2 to 12 h of onset, inflammation extends to the broader area of the peritoneum, and localized pain becomes generalized. After more than 12 h of onset, patients become hemodynamically unstable, and death may be a consequence [1]. As a result, several clinical features, such as diffuse abdominal pain, muscular defense and a change in symptoms are unique for PPU [2]. Therefore, we should distinguish PPU from PUD.

Early detection and rapid intervention are crucial for treating PPU and affecting prognosis of patients. PPU is a rare disease that only occurs in 2% to 10% of patients with PUD [3], therefore PPU is not a top of differential diagnosis of acute abdomen, leading to delay of intervention. In fact, time of intervention is one of the known prognostic values, known as the Boey score. Boey et al. stated that a delay of more than 24 h in diagnosis and management greatly worsened predicted outcomes and increased post-operative complications [4, 5]. And, prognosis itself is poor; A study prior to 1997 reported the mortality rate of PPU as 12% [6], and even a recent review reported mortality rate for PPU ranged from 1.3% to 20% [1]. Possible reasons for high mortality are pan peritonitis and consequences of bacterial infection following PPU. Perforation allows gastric contents into the peritoneal cavity, initiating chemical peritonitis, and if left untreated, bacterial contamination, intra-abdominal abscess formation and sepsis are results of continuous leakage [7]. Another reason for high mortality is delayed treatment. Therefore, misdiagnosis and delay of treatment must be prevented.

Although diagnostic tests are powerful, PPU is still overlooked due to its low incidence. The most effective method for detecting PPU is contrasted CT scan. Existence of intraperitoneal free gas is known as a sign of perforation, and both specificity and accuracy are high. In fact, by utilizing multi-detector CT (MDCT), it is possible to diagnose with accuracy as high as 98% [8] and determine the site of perforation with 86% accuracy [9]. However, from the perspective of medical costs, availability and radiation exposure, we should not perform a CT scan for all patients with acute abdominal pain. Erect chest X-ray and abdominal X-ray are useful tests from the perspectives of convenience and speed, but their clinical significance is in doubt [1, 10]. Misdiagnosis and delayed intervention for more than 24 h are important factors affecting the prognosis of PPU, thus re-evaluation of the prediction factors for PPU will allow us to suspect the disease, conduct a CT scan and diagnose PPU immediately with an appropriate procedure.

The aim of this study was to evaluate the prediction factors and develop a prediction rule for PPU.

## Methods

### Study design and settings

A retrospective case control study was conducted between August 2004 and March 2016 at St. Luke’s International Hospital in Tokyo, Japan. St. Luke’s international Hospital is a general and teaching hospital in Tokyo, Japan. Approximately 2550 outpatients are coming daily and the hospital provides 539 beds in total. All patients who were diagnosed with PPU were differentiated into the case group. In contrast, patients who had the same demographics as the case group and were diagnosed with other diseases by CT scan were referred as the control group. We evaluated prediction factors for PPU by comparing these cases and controls. St. Luke’s ethical committee approved this study (approval number: 16-J002). Since this is a retrospective study, a written consent is waived by an IRB and is deemed unnecessary. However, those who demanded not to participate in this study by looking our study notification on website were not included.

### Diagnoses of diseases

Diagnoses of PPU and other diseases were made by CT scan. All results were confirmed by board certificated radiologists. The diagnoses among those who were treated with invasive procedures such as operations were supported with findings from the procedures. Clinical decisions by emergency medicine doctors, internists or surgeons also supported the diagnoses.

### Cases and controls

We included all patients who were diagnosed with PPU in the department of emergency or internal medicine in the case group. In contrast, we considered those patients who were subjected to an abdominal CT scan at dates close to the scans of case subjects but were diagnosed with diseases other than PPU and had the same demographics, including exact age and gender, as potential control subjects. Two control subjects were randomly recruited for each case subject.

### Data collection

We obtained following patient data from electronic charts. Age, gender, and social history were obtained as demographic data. History of the present illness, past conditions and comorbidities such as PUD, gastroesophageal reflux disease, Crohn’s disease, ulcerative colitis, *H. pylori* infection, hypertension, diabetes mellitus, dyslipidemia and among other things were collected. Physical examinations, Medications, and findings of vital signs and laboratory measures such as location of pain, loss of appetite, vomiting, nausea, hematemesis, diarrhea, muscular defense, use of antiplatelet, anticoagulant, NSAIDs proton pump inhibitor, spironolactone, steroid, white blood cell count, hemoglobin, platelet count, total bilirubin, AST, C-reactive protein, etc. were also evaluated.

### Statistical analyses

First, we compared baseline characteristics between cases and controls using a chi-squared test and t-test. Then, multivariate analyses were conducted. To confirm our results, we performed the following four multivariate analyses: logistic regression with variables that had a *p* value of less than 0.2 through univariate analyses or clinically important variables; logistic regression with stepwise methods (significance level for entry: 0.05, for removal: 0.10); and conditional logistic regressions with the same two methods. Four different models are used because this is an exploratory study. Use of only one model may arise an incidental significance. In order to adjust covariance and confirm consistent results, sensitive analysis is conducted. Based on the calculated probability for each subject, ROC curves were drawn and areas under the curves (AUCs) were calculated. All analyses were performed using SPSS 19.0 (IBM, Tokyo).

## Results

A total of 408 patients were included in this study; 136 were case subjects and 272 were control subjects. Tables 1, 2, 3, and 4 shows the results obtained by univariate analyses, and many factors showed statistical significance. Among the significant factors included taking NSAIDs regularly, having a history of PUD and *H. pylori* infection and showing diffuse abdominal pain with muscular defense.

**Table 1:** Comparison of the baseline patients’ characteristics, including demographics and medical histories, between those with perforation and those without perforation by univariate analysis

	Case subjects (those with perforation)	Control subjects (those without perforation)	*p*-value
	*n* = 136	*n* = 272	
Patients’ demographics			
Age^a^, year (SD)	56.2 (16.5)	56.2 (16.5)	1.00
Male^a^, n (%)	116 (85.3)	232 (85.3)	1.00
Smoking, n (%)	98 (72.1)	114 (41.9)	**< 0.01**
Regular alcohol consumption, n (%)	75 (55.1)	112 (41.2)	0.48
Medical history			
Gastrointestinal history			
Peptic ulcer disease, n (%)	**45 (33.1)**	**27 (9.9)**	**< 0.01**
Gastroesophageal reflux disease, n (%)	2 (1.5)	4 (1.5)	1.00
Crohn’s disease, n (%)	0 (0.0)	0 (0.0)	N/A
Ulcerative colitis, n (%)	1 (0.7)	1 (0.4)	1.00
*H. pylori* infection, n (%)	**9 (6.6)**	**1 (0.4)**	**< 0.01**
Adnominal operation, n (%)	38 (27.9)	74 (27.2)	0.91
Prior ESD^b^, n (%)	1 (0.7)	1 (0.4)	1.00
Other history			
Hypertension, n (%)	34 (25.0)	68 (25.0)	1.00
Diabetes mellitus, n (%)	19 (14.0)	32 (11.8)	0.53
Dyslipidemia, n (%)	12 (8.8)	42 (15.4)	0.07
Cancer baring, n (%)	17 (12.5)	31 (11.4)	0.75
Under chemotherapy, n (%)	8 (5.9)	8 (2.9)	0.18
Chronic obstructive pulmonary disease, n (%)	**0 (0.0)**	**9 (3.3)**	**0.03**
Cirrhosis, n (%)	2 (1.5)	3 (1.1)	1.00

^a^Age and Male were matched

^b^ESD represents endoscopic submucosal dissection

Numbers in bold indicate that the *p* value is less than 0.05

**Table 2:** Comparison of baseline patients’ characteristics, including physical findings and symptoms, between those with perforation and those without perforation by univariate analysis

	Case subjects (those with perforation)	Control subjects (those without perforation)	*p*-value
	*n* = 136	*n* = 272	
Physical finding & Symptom			
Location of pain			
Abdominal pain			**< 0.01**
No pain, n (%)	13 (9.6)	94 (34.6)	
Upper abdomen, n (%)	59 (43.4)	82 (30.1)	
Mid abdomen, n (%)	9 (6.6)	39 (14.3)	
Lower abdomen, n (%)	11 (8.1)	47 (17.3)	
**Multiple abdomen**, n (%)	**44 (32.4)**	**10 (3.7)**	
Chest pain, n (%)	2 (1.5)	14 (5.1)	0.10
Back pain, n (%)	15 (11.0)	22 (8.1)	0.36
Other Associated Symptoms			
**Loss of appetite**, n (%)	**33 (24.3)**	**39 (14.3)**	**0.02**
Vomiting, n (%)	42 (30.9)	82 (30.1)	0.91
Nausea, n (%)	51 (37.5)	81 (29.8)	0.12
Hematemesis, n (%)	10 (7.4)	8 (2.9)	0.07
Constipation, n (%)	11 (8.1)	16 (5.9)	0.40
**Diarrhea**, n (%)	**9 (6.6)**	**49 (18.0)**	**< 0.01**
Hematochezia/melena, n (%)	24 (17.6)	38 (14.0)	0.38
Dizziness, n (%)	8 (5.9)	21 (7.7)	0.55
Perspiration, n (%)	20 (14.7)	34 (12.5)	0.54
Pallor, n (%)	8 (5.9)	12 (4.4)	0.63
Respiratory distress, n (%)	9 (6.6)	17 (6.3)	1.00
**Muscular defense**, n (%)	**93 (68.4)**	**25 (9.2)**	**< 0.01**

Numbers in bold indicate that the *p* value is less than 0.05

**Table 3:** Comparison of baseline patients’ medications between those with perforation and those without perforation by univariate analysis

	Case subjects (those with perforation)	Control subjects (those without perforation)	*p*-value
	*n* = 136	*n* = 272	
Medications			
Antiplatelet, n (%)			0.78
None, n (%)	127 (93.4)	256 (94.1)	
Aspirin, n (%)	6 (4.4)	11 (4.0)	
Clopidogrel, n (%)	0 (0.0)	1 (0.4)	
Limaprost alfadex, n (%)	0 (0.0)	1 (0.4)	
Dual Antiplatelet Therapy, n (%)	3 (2.2)	3 (1.1)	
Anticoagulant, n (%)	7 (5.1)	9 (3.3)	0.42
**NSAIDs daily**, n (%)	**15 (11.0)**	**13 (4.8)**	**0.02**
NSAIDs rescue, n (%)	11 (8.1)	16 (5.9)	0.40
Proton pump inhibitor, n (%)	10 (7.4)	21 (7.7)	1.00
H_2_ blocker, n (%)	1 (0.7)	10 (3.7)	0.11
Bisphosphonate, n (%)	4 (2.9)	3 (1.1)	0.23
Spironolactone, n (%)	1 (0.7)	7 (2.6)	0.28
Steroid, n (%)	5 (3.7)	8 (2.9)	0.77

Numbers in bold indicate that the *p* value is less than 0.05

**Table 4:** Comparison of baseline patients’ characteristics, including vital signs and laboratory data, between those with perforation and those without perforation by univariate analysis

	Case subjects (those with perforation)	Control subjects (those without perforation)	*p*-value
	*n* = 136	*n* = 272	
Vital signs			
Systolic blood pressure (SD)	130.4 (24.0)	133.5 (28.2)	0.39
Diastolic blood pressure (SD)	74.1 (18.5)	74.3 (16.4)	0.94
**Respiratory rate** (SD)	**20.2 (6.1)**	**18.2 (5.2)**	**0.01**
Heart rate (SD)	81.2 (15.8)	80.7 (16.3)	0.82
Body temperature (SD)	36.6 (0.91)	36.7 (0.97)	0.27
Laboratory data (SD)			
**White blood cell count** (SD)	**10.7 (6.5)**	**9.2 (5.1)**	**0.01**
**Hemoglobin** (SD)	**12.4 (4.8)**	**13.5 (3.2)**	**0.02**
**Platelet count** (SD)	**220.0 (130.0)**	**175.5 (101.1)**	**< 0.01**
**Total protein** (SD)	**6.8 (1.0)**	**7.1 (0.8)**	**0.02**
**Albumin** (SD)	**3.8 (0.9)**	**4.0 (0.7)**	**0.04**
Blood urea nitrogen (SD)	19.7 (15.0)	17.1 (13.1)	0.10
Creatinine (SD)	1.0 (0.9)	1.0 (1.2)	0.81
Total bilirubin (SD)	0.8 (0.5)	1.0 (1.2)	0.06
**AST** (SD)	**23.6 (25.1)**	**47.1 (114.3)**	**< 0.01**
**ALT** (SD)	**21.2 (18.5)**	**40.2 (76.8)**	**< 0.01**
Sodium (SD)	112.7 (50.9)	121.3 (43.8)	0.11
Potassium (SD)	3.9 (0.5)	4.0 (0.5)	0.33
Chlorine (SD)	27.5 (30.3)	25.3 (30.0)	0.50
C-reactive protein (SD)	4.0 (8.2)	3.2 (5.0)	0.32
APTT (SD)	25.9 (7.5)	26.7 (9.2)	0.39
PT (SD)	8.8 (5.4)	10.0 (6.5)	0.11

Numbers in bold indicate that the *p* value is less than 0.05

Table 5 shows the results from the multivariate analyses. Four different models were conducted and significant differences were found for muscular defense and PUD in all models. The data are as follows for the symptom of muscular defense: logistic step-wise model (odd ratio [OR]: 366.2, 95% confidence interval (CI): 61.9–2166.2), conditional step-wise model (OR: 76.6, 95% CI: 13.7–429.6), logistic forced-entry model (OR: 28.4, 95% CI: 11.6–69.6), and conditional forced-entry model (OR: 23.8, 95% CI: 5.70–100.0). The data are as follows for a history of PUD: logistic step-wise model (OR: 7.81, 95% CI: 1.98–30.8), conditional step-wise model (OR: 21.9, 95% CI: 3.92–122.5), logistic forced-entry model (OR: 3.67, 95% CI: 1.47–9.18), and conditional forced-entry model (OR: 6.40, 95% CI: 1.13–36.2). The following seven factors were statistically significant in two to three models: diffuse pain, daily NSAID use, antiplatelet use, back pain, vomiting, platelet count, and hemoglobin concentration. Furthermore, anticoagulant, proton pump inhibitor (PPI) and steroid medications were found as irrelevant to upper gastric perforation in this study.

**Table 5:** Risk factors for perforation from multivariate logistic regression and sensitivity analysis

	Odds Ratios (95% Confidence Interval)			
	Stepwise		Forced-entry	
Social History	Logistic	Conditional	Logistic	Conditional
Smoking			1.02	1.39
			(0.45–2.31)	(0.42–4.64)
Alcohol			0.68	0.56
			(0.31–1.50)	(0.19–1.62)
Past Medical History				
PUD	**7.81**	**21.9**	**3.67**	**6.40**
	**(1.98–30.8)**	**(3.92–122.5)**	**(1.47–9.18)**	**(1.13–36.2)**
GERD	**256.7**			
	**(12.5–5274.1)**			
Medications				
NSAID daily use	**28.5**		**4.71**	5.40
	**(3.00–270.3)**		**(1.29–17.2)**	(0.55–53.5)
NSAID rescue use	**0.05**			
	**(0.004–0.57)**			
Antiplatelet	**5.90**		**2.37**	3.20
	**(2.59–13.4)**		**(1.29–4.34)**	(0.75–13.6)
Anticoagulant			1.04	0.85
			(0.13–8.41)	(0.03–22.3)
PPI*			0.50	2.03
			(0.13–1.86)	(0.24–16.9)
Physical Findings				
Location of pain No pain Reference				
Upper abdomen	**4.40**		1.90	2.61
	**(1.10–17.7)**		(0.71–5.12)	(0.59–11.5)
Mid abdomen	2.20		1.23	2.47
	(0.28–16.6)		(0.28–5.45)	(0.13–46.6)
Lower abdomen	1.62		0.83	2.18
	(0.27–9.81)		(0.21–3.23)	(0.33–14.4)
Multiple abdomen	**287.7**		**39.4**	**51.8**
	**(22.9–3619.8)**		**(8.79–176.3)**	**(3.57–752.9)**
Muscular defense	**366.2**	**76.6**	**28.4**	**23.8**
	**(61.9–2166.2)**	**(13.7–429.6)**	**(11.6–69.6)**	**(5.70–100.0)**
Back pain	**24.4**	**100.7**		
	**(3.30–180.6)**	**(7.05–1438.0)**		
Chest pain	**0.002**			
	**(0.00–0.12)**			
Loss of appetite			**3.63**	3.43
			**(1.40–9.46)**	(0.75–15.7)
Hematemesis	**141.2**			
	**(7.02–2838.0)**			
Diarrhea	**0.19**			
	**(0.04–0.89)**			
*H. Pylori* infection	**1522.0**			
	**(14.4–160,502.9)**			
Vomiting	**0.03**		**0.39**	0.42
	**(0.01–0.16)**		**(0.18–0.88)**	(0.12–1.44)
Nausea	**9.69**			
	**(2.27–41.4)**			
Laboratory Data				
C-Reactive Protein	**1.13**		1.04	1.02
	**(1.05–1.22)**		(1.00–1.10)	(0.95–1.11)
Platelet counts			**1.00**	**1.01**
			**(1.01–1.01)**	**(1.00–1.01)**
Hemoglobin	**0.75**	**0.78**		
	**(0.65–0.87)**	**(0.66–0.92)**		
Total bilirubin	**0.32**			
	**(0.16–0.68)**			
AST	**0.97**			
	**(0.95–1.00)**			
Creatinine		**1.67**		
		**(1.10–2.53)**		

Numbers in bold indicate that the *p* value is less than 0.05

**Fig. 1:**
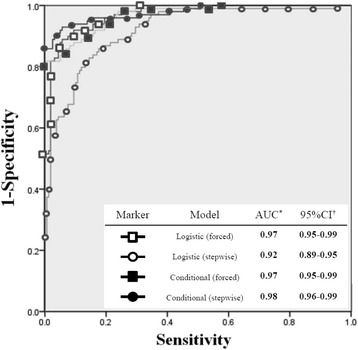
The comparison of receiver operating characteristic curves (ROCs) models between four different multivariate analyses. Figure legends: ROCs of four models are shown above and area under the curves (AUCs) and 95% confidence interval (CI) are also calculated. Both AUCs and 95% CI from all models are high enough to confirm the results. Especially, AUC of conditional step-wise method shows the highest value, indicating its high reliability

Figure 1 shows ROCs of the four models. The AUCs for each model are as follows: logistic forced-entry method: 0.97 (95% CI: 0.95–0.99), logistic step-wise model: 0.92 (95% CI: 0.89–0.95), conditional forced-entry model: 0.97 (CI: 0.95–0.99), and conditional step-wise method: 0.98 (95% CI: 0.96–0.99). The AUCs of all models were high enough to confirm the results.

## Discussion

Our prediction models exhibited many different predicted risk factors for PPU and outcomes of the AUCs verified their significance. Some were similar to the known prediction factors of PUD, but some were different.

Some similarity among prediction factors of PUD and PPU was reconfirmed though our study. According to prior studies, the following factors increased the OR for PUD: NSAID use (OR: 4.85), antiplatelet drug use (OR: 1.68) and *H. pylori* infection (OR: 18.1) [11, 12]. Furthermore, PUD is known to present nausea, hematemesis, and loss of appetite and is often seen in patients with gastroesophageal reflux disease (GERD) and a previous history of ulcer. In addition, when symptoms such as persisting radiating back pain and sudden rapidly exacerbating multiple abdominal pains were associated, potential complications, including perforation, were suggested [13]. As well, in our PPU study, the above factors were shared prediction factors for perforation. We hypothesized that patients with GERD and/or a previous history of ulcer were susceptible to gastric complications by nature, but even without the nature of susceptibility, NSAIDs or antiplatelet drugs alone could penetrate the gastric mucous membrane by inhibiting cyclooxygenase-1 (COX-1). We concluded that symptoms such as nausea, hematemesis, loss of appetite, radiating and multiple pains resulted from a wide range of mucous damage and stimulation of the nerves distributed throughout the peritoneum and mesentery [14].

There were some differences in the prediction factors between PUD and PPU. For one, anticoagulants were thought of as a prediction factor for PUD, increasing the OR to 1.98 [15], but anticoagulants did not increase the risk of PPU in our study. Certainly, in molecular biological point of view, coagulation is a defense mechanism that can protect gastric mucous membrane, and by the use of anticoagulants, homeostasis of reproductive process can be disrupted. However, in clinical setting, by the perspective of our findings we consider that anticoagulants alone do not cause significant influence on biological setting. We hypothesized that the insignificance of anticoagulant drugs was due to their mechanism. Unlike Cox-1 inhibitors, anticoagulants are medications which inhibit the productions of fibrin at the end of the blood coagulation reaction [16]_;_ they may promote the bleeding risk, but may not cause damage to the gastric mucous membrane. In addition, vomiting is a symptom often seen in patients with PUD and with esophageal perforation [17], but a reduction in vomiting was statistically significant in PPU subjects. We hypothesized that continuous leakage reduced the gastric contents, leading to less vomiting as a physical symptom. Finally, an increase in platelet count was statistically significant in PPU, and could be used as a prediction factor. Platelets are component of blood, necessary to arrest bleeding, thus thrombocytopenia is known cause of bleeding. In our PPU study, however, platelet counts were significantly increased. We presumed that peritonitis caused inflammation and perforation stimulated platelet aggregation; reactive thrombocytosis is a phenomenon seen in the PPU patients.

There are some limitations in our study. Our study is a research at a single institution and targets only one ethic group. Thus, generalization of our outcome into different ethnicity might be difficult and these could be a selection bias. Moreover, there are some missing data because initial encounter with patients and diagnostic process are conducted by different doctors each time. For example, it could be an interesting research by comparing X-ray and CT scan to patients with abdominal pain. Since X-ray is not gold standard for diagnosing acute abdomen, thus unperformed each case, we could not gather enough data for X-ray. Furthermore, there may be a group of false negative. Some outpatients with abdominal disturbance may not be taken CT scan, and may not be diagnosed as PPU. However in our hospital, CT scan is available 24 h every day, every year, so that barrier for taken CT scan is very low. In addition to the low barrier, board certificated radiologists are within reach at all times, false negative is limited. Moreover, the phenomenon which some variables are significant in multivariate analyses but not significant in univariate analysis is mainly coming from confounding factors. In addition, the effect of unbalanced sample size, the influence of missing data and an extremely large within-group variation, relative to between-group variation are other variables, contributing to the discrepancy.

## Conclusions

In this study, out of many categories from past medical histories, including physical findings, medications, and laboratory data, prediction factors for upper gastrointestinal perforation were extracted. In the clinical setting, even though perforation is a rare disease, when the patients present with muscular defense and PUD history, a physician should take abdominal CT scan without any hesitation for diagnosing PPU.

## Abbreviations

AUC: Area under the curve
CI: Confidence interval
COX-1: Cyclooxygenase-1
GERD: Gastroesophageal reflux disease
MDCT: Multi-detector CT
OR: Odds ratio
PPI: Proton pump inhibitor
PPU: Perforated peptic ulcer
PUD: Peptic ulcer disease
ROCs: Receiver operating characteristic curves
